# Supplementary material for: Co-expression of a PD-L1-specific chimeric switch receptor augments the efficacy and persistence of CAR T cells via the CD70-CD27 axis
Source: Nat Commun. 2022 Oct 13;13:6051. doi: 10.1038/s41467-022-33793-w (PMC9561169; doi:10.1038/s41467-022-33793-w)
Supplement: Supplementary file 1 — Supplementary information [file 41467_2022_33793_MOESM1_ESM.pdf]

Co-expression of a PD-L1-specific chimeric switch receptor augments the efficacy and persistence of CAR T cells via the CD70-CD27 axis

Le Qin, Peng Li et al.

Supplementary Information

Supplementary Figure 1-10

Supplementary Table 1-2

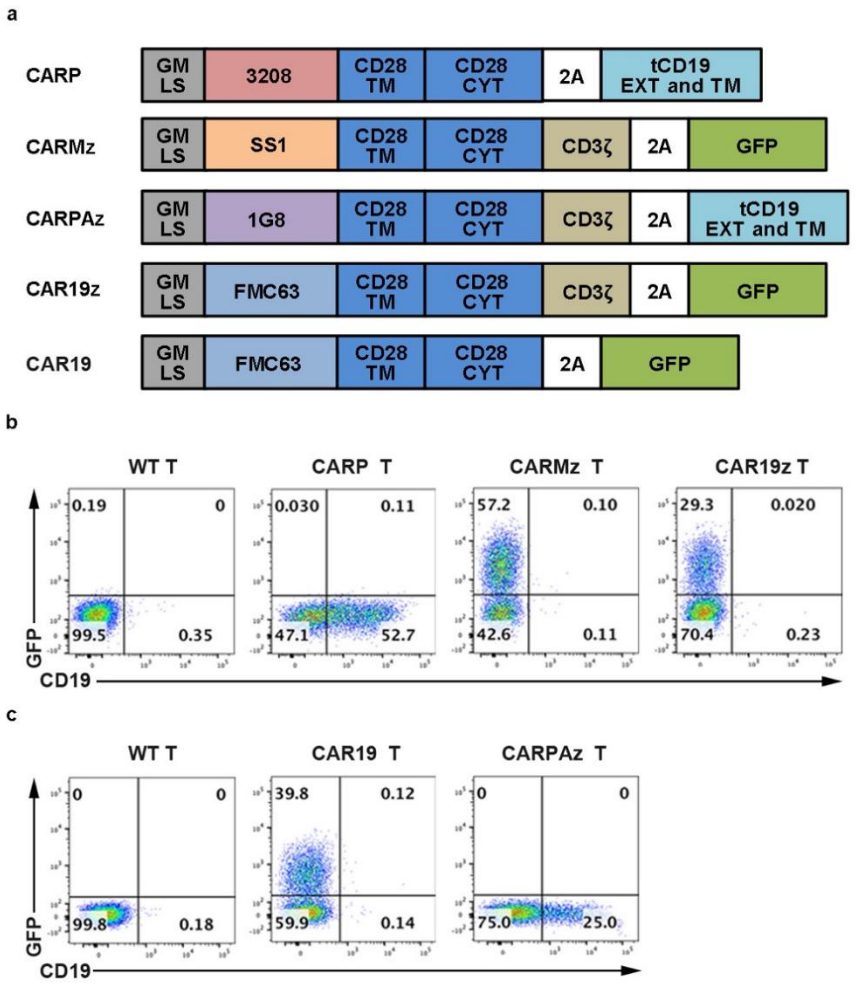

Supplementary Figure 1. Schematic diagrams of the CAR constructs. **a** CARP contains a 3208 scFv that binds human PD-L1 and CAR19 contains a FMC63 scFv that targets human CD19, both of them carry the CD28 transmembrane and cytoplasmic domain without the CD3ζ chain. CAR19z has the same structure as CAR19 except that it contains the CD3ζ chain, CAR19z is used as a negative control in this study. CARMz includes a SS1 scFv that binds human MSLN and CARPAz contains a 1G8 scFv that targets human PSCA. The extracellular and transmembrane domains of CD19 (truncated CD19, tCD19) were added to CARP and CARPAz as a reporter gene. LS: leader sequence, EXT: extracellular domain, TM: transmembrane domain, CYT: cytoplasmic domain. **b-c** Representative transduction efficiencies of CARMz, CARP, CAR19z, CAR19 and CARPAz.

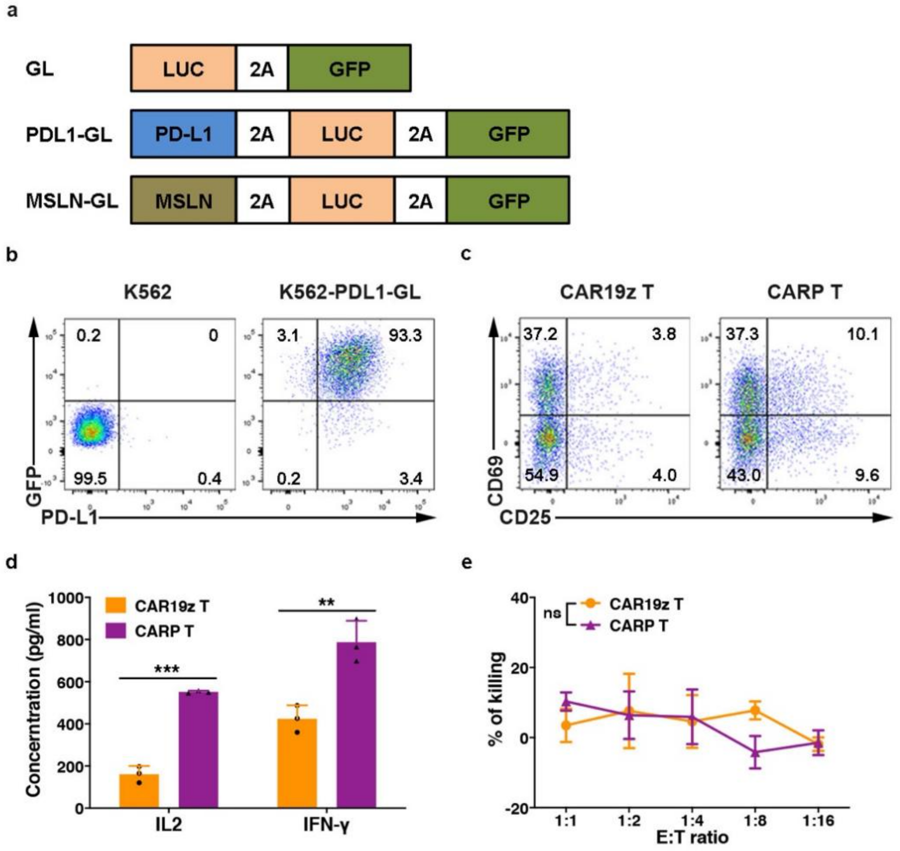

Supplementary Figure 2. Enhanced activation but not cytotoxicity of CARP T cells is induced by coculture with PD-L1-positive tumor cells. a Schematic diagram of the GL, PDL1-GL and MSLN-GL vectors. LUC: luciferase, MSLN: mesothelin. **b** Percentages of PD-L1 and GFP double-positive cells in K562 and K562-PDL1-GL cells. **c** Percentages of CD25 and CD69 double-positive cells in CARP T and control CAR19z T cells after coculture with K562-PDL1-GL cells at a 1:1 E:T ratio for 24 h (gated on CAR^+ cells). **d** The production of IL-2 and IFN-γ by CARP T and control CAR19z T cells after coculture with K562-PDL1-GL cells. Data are presented as mean ± SD (N=3 technical repeats). *p* Values were calculated by two-side unpaired t-test (CARP T vs. CAR19z T=IL2: 6.619E-05, IFN-γ: 0.006). **e** CARP T and control CAR19z T cells cytotoxicity against K562-PDL1-GL cells were measured at various E:T ratios. Data are presented as mean ± SD (N=3 technical repeats). *p* Values were calculated by two-way ANOVA with Sidak's multiple comparisons test (CARP T vs. CAR19z T=0.999). ** $p<0.01$, and *** $p<0.001$.

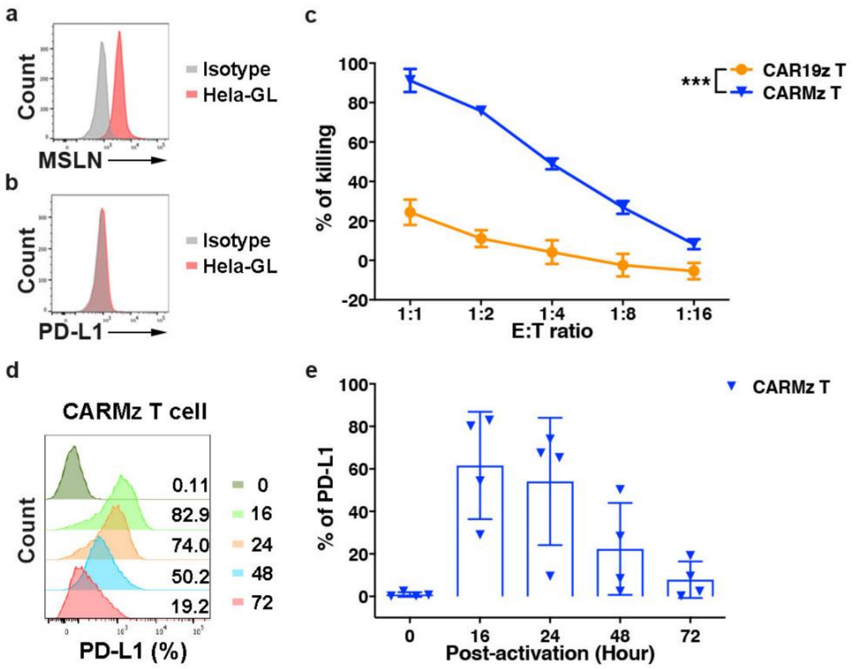

Supplementary Figure 3. CARMz T cells upregulate PD-L1 post-coculture with HeLa-GL cells. a-b The expression of MSLN and PD-L1 in HeLa-GL cells. **c** CARMz T and CAR19z T cells cytotoxicity against HeLa-GL cells were measured at various E:T ratio. Data are presented as mean ± SD (N=3 independent experiments). *p* Values were calculated by two-way ANOVA with Sidak's multiple comparisons test (CARMz T vs. CAR19z T=7.48E-07). **d** The representative expression of PD-L1 in CARMz T cells after activation with HeLa-GL cells for 0, 16, 24, 48 and 72 h. **e** Proportion of PD-$L1^{+}$ CARMz T cells post-activated by HeLa-GL cells for 0, 16, 24, 48 and 72 h in **d**. Data are presented as mean ± SEM (N=4 biological samples). *** $p<0.001$.

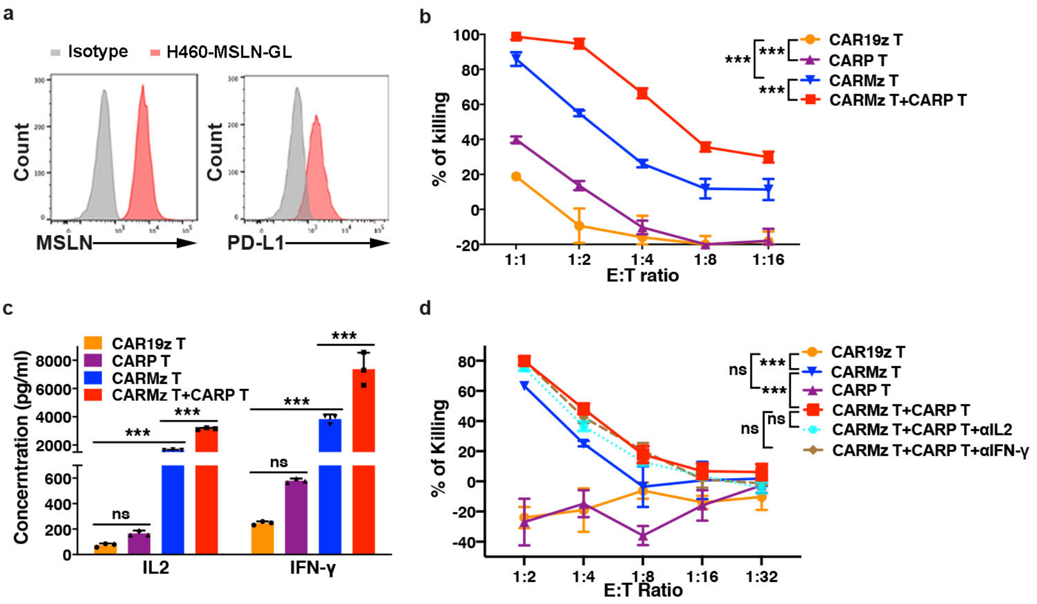

Supplementary Figure 4. CARP T cells enhance the antitumor activity of CARMz T cells to H460-MSLN-GL cells. a The expression of MSLN (left) and PD-L1 (right) in H460-MSLN-GL cells. **b** CARP T, CARMz T, a mixture of CARMz T and CARP T and control CAR19z T cells cytotoxicity against H460-MSLN-GL were measured at various E:T ratio. *p* Values (CARP T vs. CAR19z T=1.499E-04, CARMz T vs. CAR19z T=4.71E-13, CARMz T+CARP T vs. CARMz T=4.72E-13). **c** The bar chart shows IL-2 and IFN-γ secretion by CARP T, CARMz T, a mixture of CARMz T and CARP T and CAR19z T cells after coculture with H460-MSLN-GL. *p* Values were calculated by one-way ANOVA with Sidak's post hoc test (CARP T vs. CAR19z T=IL2: 0.051, IFN-γ: 0.987, CARMz T vs. CAR19z T=IL2: 4.25E-11, IFN-γ: 5.284E-04, CARMz T+CARP T vs. CARMz T=IL2: 7.066E-11, IFN-γ: 5.746E-04). **d** CARP T, CARMz T, a mixture of CARMz T and CARP T, a mixture of CARMz T and CARP T treated with anti-IL2 mAb (αIL2, 10 μg/ml), a mixture of CARMz T and CARP T treated with anti-IFN-γ mAb (αIFN-γ, 10 μg/ml) and control CAR19z T cells cytotoxicity against Hela-GL cells were measured at various E:T ratios. *p* Values (CARP T vs. CAR19z T=0,474, CARMz T vs. CAR19z T=1.99E-11, CARMz T+CARP T vs. CARMz T=7,368E-06, CARMz T+CARP T+αIL2 vs. CARMz T+CARP T=0.083, CARMz T+CARP T+αIFN-γ vs. CARMz T+CARP T=0.818). Data of **b**, **c** and **d** are presented as mean ± SD (N=3 technical repeats). *p* Values of **b** and **d** were calculated by two-way ANOVA with Tukey's multiple comparisons test. ** *p*<0.01, and *** *p*<0.001.

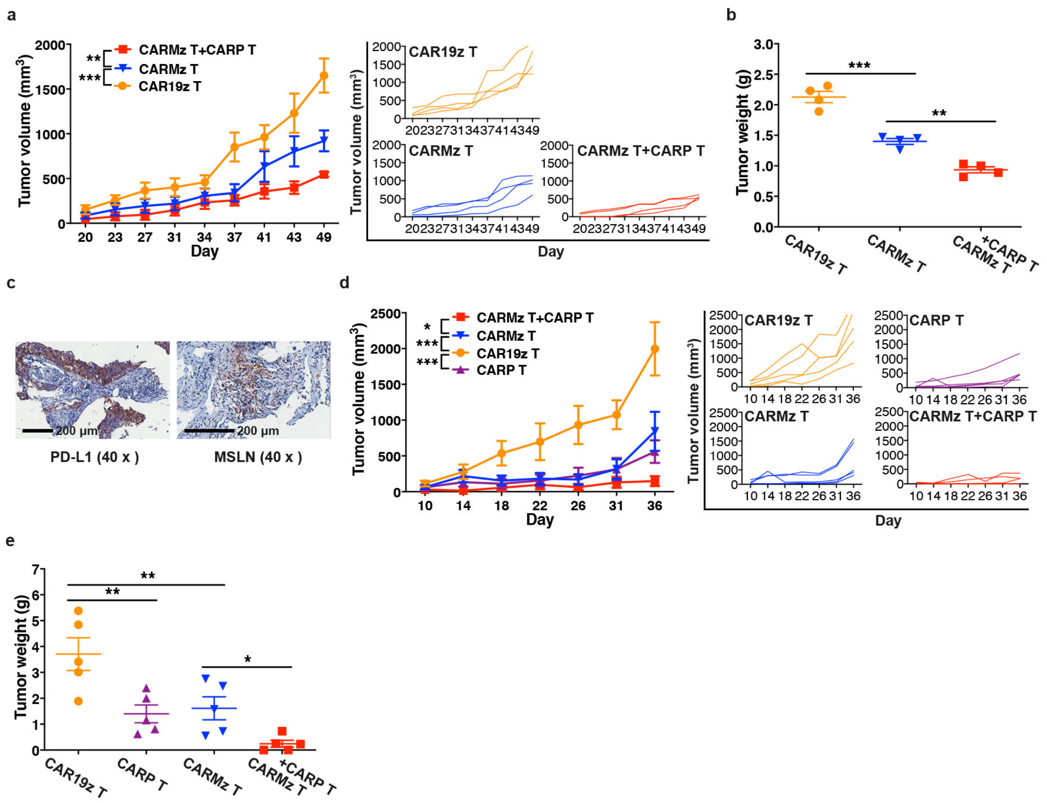

Supplementary Figure 5. CARP T cells enhanced the *in vivo* antitumor efficacy of CARMz T cells. a-b NSI mice bearing HeLa-GL tumors (5×10^5, established for 20 days) were infused with CARMz T, a mixture of CARMz T and CARP T or CAR19z T cells (5×10^6). **a** Tumor volumes were monitored on the indicated days. Individual tumor responses to CAR-T cell injection are shown with spider plots on the left. *p* Values (CAR19z T vs. CARMz T=1.254E-07, CARMz T+CARP T vs. CARMz T=0.002). **b** Tumor weights were measured after mouse euthanasia. *p* Values (CAR19z T vs. CARMz T=8.499E-05, CARMz T+CARP T vs. CARMz T=0.002). **c** The expression of PD-L1 and MSLN in primary NSCLC sample were detected by immunohistochemistry. **d-e** NSI mice bearing MSLN and PD-L1 double-positive NSCLC tumors (established for 10 days) were infused with CARP T, CARMz T, a mixture of CARMz T and CARP T or CAR19z T cells (5×10^6). **d** Tumor volumes were monitored on the indicated days. Individual tumor responses to CAR-T cell injection are shown with spider plots on the left. *p* Values (CAR19z T vs. CARP T=4.988E-11, CAR19z T vs. CARMz T=1.974E-09, CARMz T+CARP T vs. CARMz T=0.045). **e** Tumor weights were measured after mouse euthanasia. *p* Values (CAR19z T vs. CARP T=0.001, CAR19z T vs. CARMz T=0.003, CARMz T+CARP T vs. CARMz T=0.037). Data of **a** and **b** are presented as mean ± SEM (N=4 mice per group). Data of **d** and **e** are presented as mean ± SEM (N=5 mice per group). *p* Values of **a** and **d** were calculated by two-way ANOVA with Tukey's multiple comparisons test. *p* Values of **b** and **e** were calculated by one-way ANOVA with Sidak's post hoc test. * $p<0.05$, ** $p<0.01$, and *** $p<0.001$.

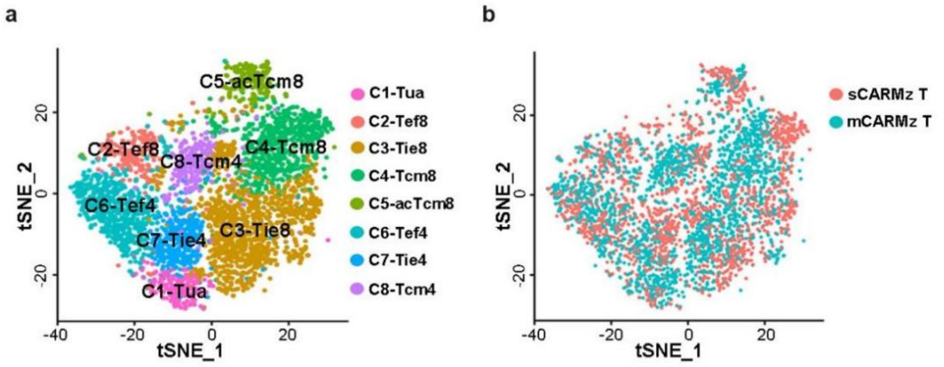

Supplementary Figure 6. The tSNE plots of CARMz T cells from sCARMz T and mCARMz T. a The tSNE projection of single CARMz T from a mixture of CARMz T and CARP T (mCARMz T) and separated CARMz T cells (sCARMz T), showing the formation of 8 main clusters (C1-C8). C1-Tua: non-activated T cells, C2-Tef8: $CD8^+$ effector T cells, C3-Tie8: partially differentiated $CD8^+$ effector T cells, C4-Tcm8: $CD8^+$ central memory T cells, C5-acTcm8: activated $CD8^+$ central memory T cells, C6-Tef4: $CD4^+$ effector T cells, C7-Tie4: partially differentiated $CD4^+$ effector T cells, C8-Tcm4: $CD4^+$ central memory T cells. Each dot corresponds to one single cell. **b** Single cells from sCARMz T cell (red) and mCARMz T cell (blue) clusters in distinct regions of the tSNE space.

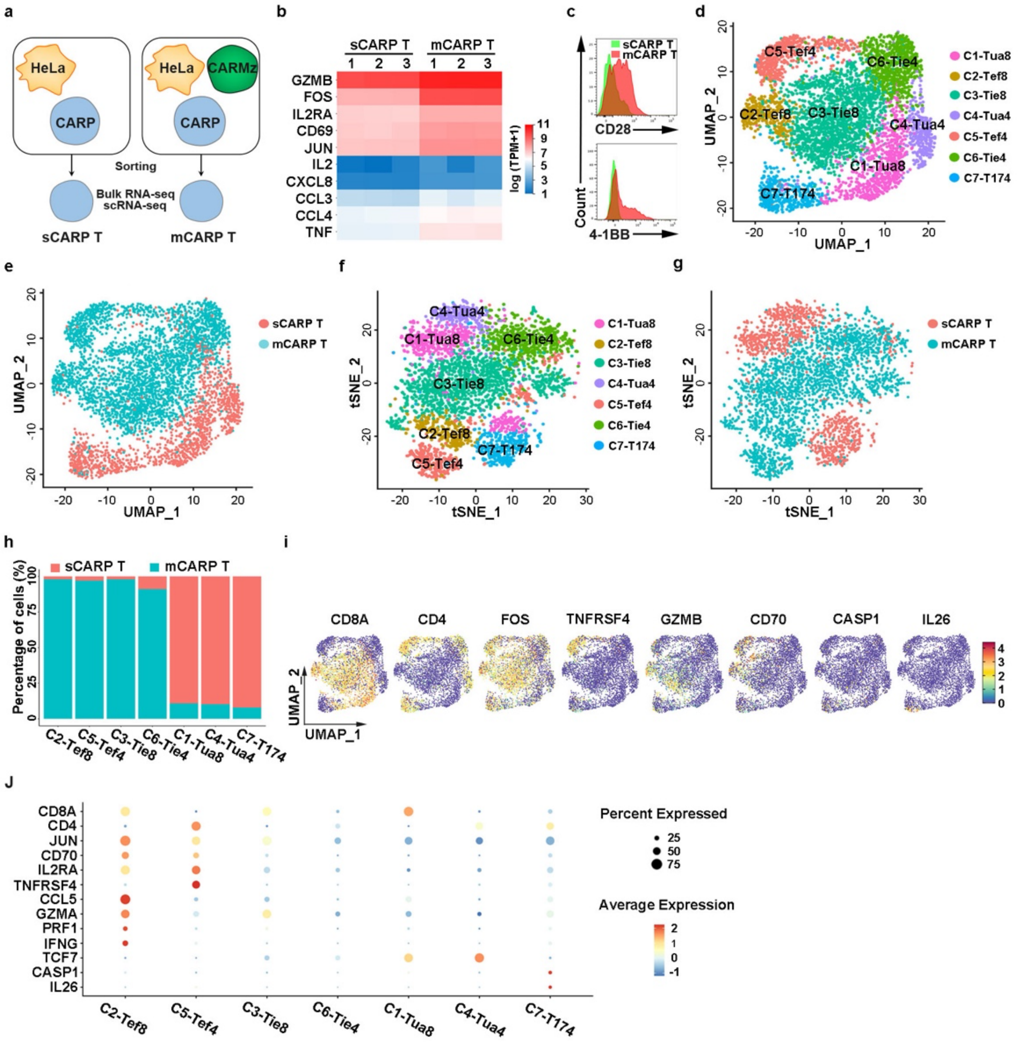

Supplementary Figure 7. RNA-seq analysis of CARP T from a mixture of CARMz T and CARP T (mCARP T) and individual CARP T cells (sCARP T) post-coculture with HeLa-GL cells. a RNA-seq strategy: CARP T cells were separated from individual CARP T (sCARP T) and a mixture of CARMz T and CARP T cells (mCARP T) by flow cytometry sorting, and then processed for bulk RNA-seq and scRNA-seq. **b** The heatmap shows clustering of DEGs between sCARP T and mCARP T cells based on bulk RNA-seq analysis (cutoff: absolute log2(fold change) ≥ 1; adjusted P value ≤0.05). Each group had three biological replicates. **c** Percentages of $CD28^{+}$ and 4-$1BB^{+}$ cells in individual CARP T (sCARP T) and CARP T from a mixture of CARMz T and CARP T cells (mCARP T) post-coculture with HeLa-GL cells at a 1:1 E:T ratio for 24 h (gated on $CD19^{+}$ cells). **d** The UMAP projection of single CARP T cells from sCARP T and mCARP T cells. **e** Single cells from sCARP T (red) and mCARP T cell (blue) cluster in distinct regions of the UMAP space. **f** The tSNE projection of single CARP T cells from sCARP T and mCARP T cells. **g** Single cells from sCARP T (red) and mCARP T cells (blue) cluster in distinct regions of the tSNE space. **h** Percentages of sCARP T and mCARP T cells in each cluster. **i** Single-cell transcript levels of CD8, CD4, FOS, TNFRSF4, GZMB, CD70, CASP1 and IL26 illustrated in UMAP plots. **j** Dot plot of selected DEGs expressed in each cluster.

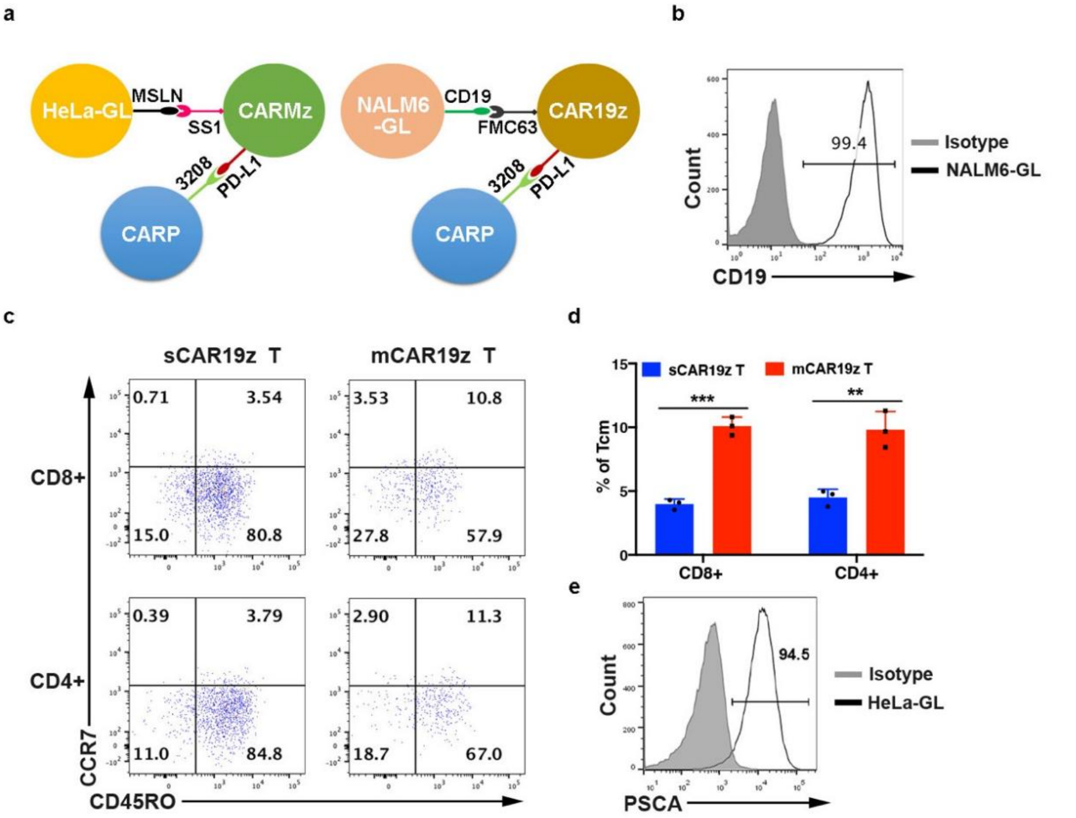

Supplementary Figure 8. CARP T cells enhance the antitumor effect of CAR19z T cells. a Schematic diagram of heterotypic binding of CARMz T to CARP T (left) and CAR19z T to CARP T cells (right). Antigens and scFvs are indicated. **b** FACS analysis of the expression of CD19 in NALM6-GL cells. **c** Representative phenotype of CAR19z T (gated on $CD8^{+}GFP^{+}$ cells and $CD4^{+}GFP^{+}$ cells) from separate CAR19z T (sCAR19z T) and a mixture of CAR19z T and CARP T cells (mCAR19z T) post-coculture with NALM6-GL cells. **d** Proportion of Tcm (central memory T cells, $CD45RO^{+}CCR7^{+}$) in sCAR19z T and mCAR19z T cells post-coculture with NALM6-GL for 36 h. Data are presented as mean ± SD (N=3 biological samples). *p* Values were calculated by two-side unpaired t-test (mCAR19z T vs. sCAR19z T=CD8+: 1.996E-04, CD4+: 0.004). **e** FACS analysis of the expression of PSCA in HeLa-GL cells.

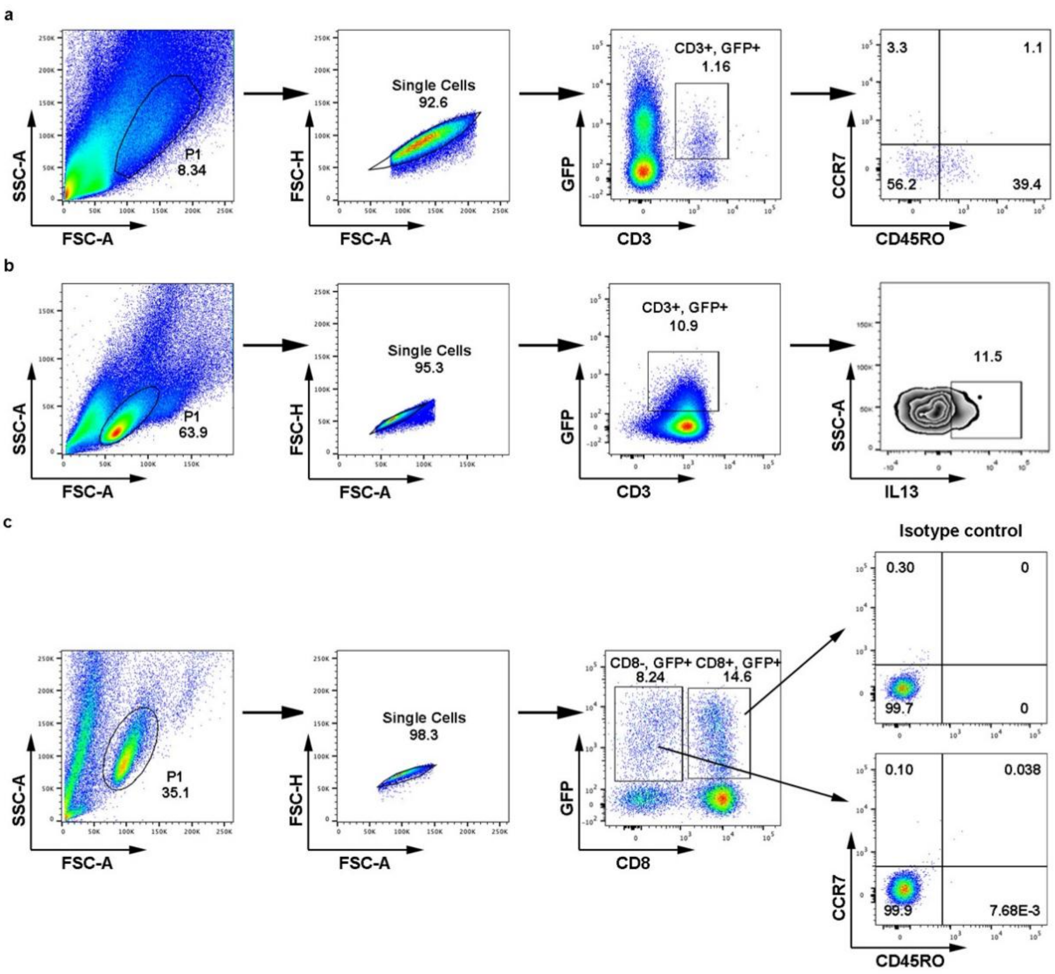

Supplementary Figure 9. FACS gating strategy 1. a. FACS gating strategy for analyzing phenotype of sCARMz T, mCARMz T and CAR19z T cells within tumors in Fig. 1e, f. **b** FACS gating strategy for measuring IL13-scecreting cells in sCARMz T, mCARMz T and CARMz T cells treated with AZ in Fig. 3f. **c** FACS gating strategy for analyzing phenotype of sCARMz T, mCARMz T, CARMz T treated with αCD70 (mCARMz T+αCD70) and control CAR19z T cells in Fig. 4i, 4j, 6e, 6f. Isotype control was used to define the gates.

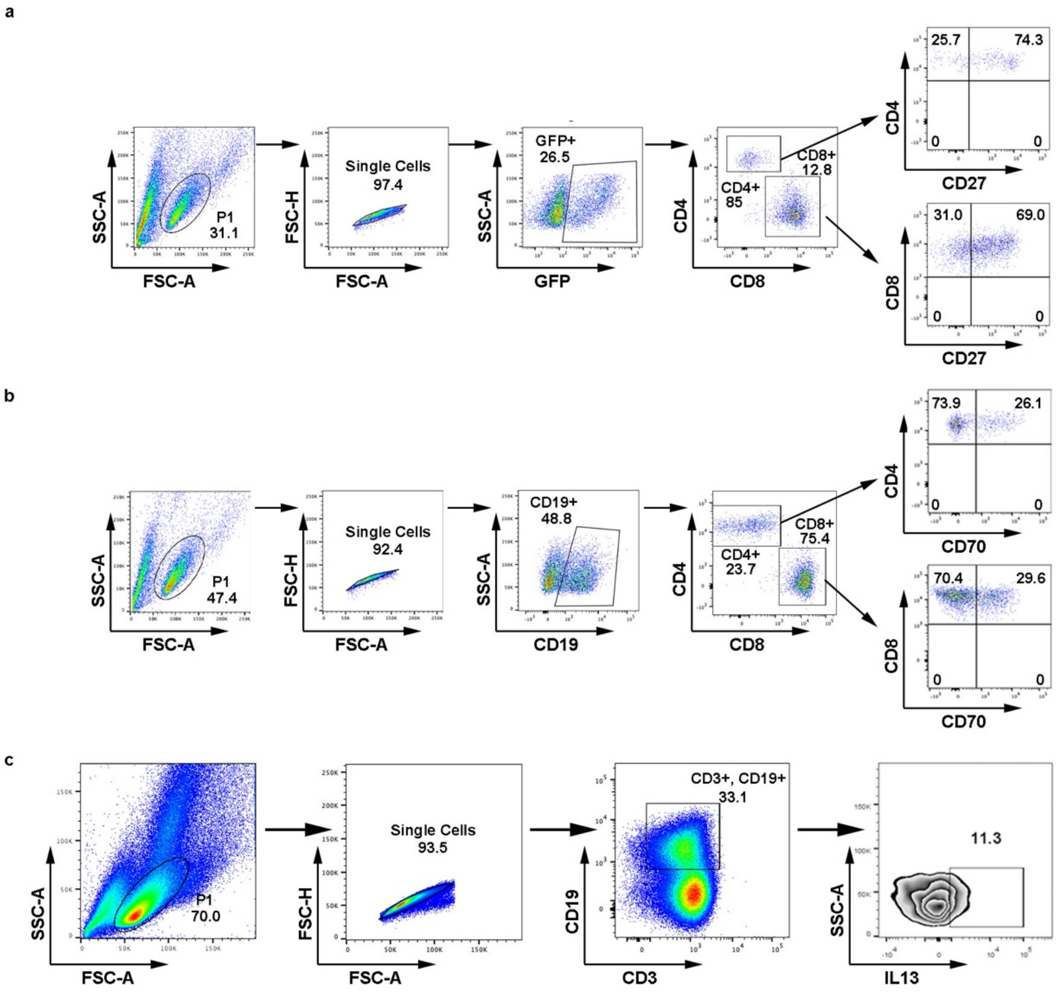

Supplementary Figure 10. FACS gating strategy 2. a. FACS gating strategy for analyzing percentage of CD27 expression in CD8$^+$ and CD4$^+$ CARMz T cells in Fig. 6a, b. **b** FACS gating strategy for analyzing percentage of CD70 expression in CD8$^+$ and CD4$^+$ CARP T cells in Fig. 6c, d. **c** FACS gating strategy for measuring IL13-sceecreting cells in sCARPAz T and mCARPAz T cells in Fig. 7c.

Supplementary Table 1. The interaction strength of M-Tcm4 and M-Tcm8 with other subsets of CARMz T and CARP T cells.

Cell cluster	M-Tcm4	M-Tcm8
M-Tcm4	0.07	0.02
M-Tcm8	1.34	1.31
M-Tef4	0.14	0.17
M-Tef8	1.5	1.38
M-Tie4	0.03	0.02
M-Tie8	1.36	1.34
M-ac-Tcm8	0.07	0.05
M-Tua	0	0
P-Tef4	0.21	0.24
P-Tef8	1.74	1.84
P-Tie4	0.04	0.02
P-Tie8	1.38	1.34
P-T174	0.11	0.06
P-Tua4	0.04	0
P-Tua8	1.77	1.76

The interaction strength of $CD4^+$ central memory-like CARMz T cells (M-Tcm4) and $CD8^+$ central memory-like CARMz T cells (M-Tcm8) with other subsets of CARMz T and CARP T cells was analyzed by CellChat tool. The interaction strength represents intercellular communication probability.

Supplementary Table 2. Antibodies for FACS used in this research.

Name	Clone	Brand	Catalog	Diluted
Anti-human CD3-PE/cyanine7	UCHT1	Biolegend	300420	5µL/test, 150µg/ml
Anti-human CD4-APC	OKT4	Biolegend	317416	5µL/test, 150µg/ml
Anti-human CD4-APC/cyanine7	OKT4	Biolegend	317418	5µL/test, 100µg/ml
Anti-human CD8-PE	HIT8a	Biolegend	344706	5µL/test, 12µg/ml
Anti-human CD8a-PerCP/cyanine5.5	HIT8a	Biolegend	300924	5µL/test, 40µg/ml
Anti-human CD28-APC	CD28.2	invitrogen	17-0289-42	5µL/test, 50µg/ml
Anti-human CD28-APC/cyanine7	CD28.2	Biolegend	302966	5µL/test, 100µg/ml
Anti-human 4-1BB-PE	4B4-1	Biolegend	309804	5µL/test, 100µg/ml
Anti-human CD19-APC	HIB19	Biolegend	302212	5µL/test, 50µg/ml
Anti-human CD25-PE	BC96	Biolegend	302606	5µL/test, 100µg/ml
Anti-human CD69-APC	FN50	Biolegend	310910	5µL/test, 100µg/ml
Anti-human PD-L1-APC	MIH2	Biolegend	393610	5µL/test, 200µg/ml
Anti-human MSLN-APC	420411	R&D	FAB32652A	10µL/test, 100µg/ml
Anti-human CD70-PE/cyanine7	113-16	Biolegend	355112	5µL/test, 100µg/ml
Anti-human CD27-PE	M-T271	Biolegend	356406	5µL/test, 100µg/ml
Anti-human IL13-PE	85BRD	invitrogen	12-7136-42	5µL/test, 25µg/ml
