# Supplementary material for: Co-expression of a PD-L1-specific chimeric switch receptor augments the efficacy and persistence of CAR T cells via the CD70-CD27 axis
Source: Nat Commun. 2022 Oct 13;13:6051. doi: 10.1038/s41467-022-33793-w (PMC9561169; doi:10.1038/s41467-022-33793-w)
Supplement: Supplementary file 3 — Reporting Summary [file 41467_2022_33793_MOESM3_ESM.pdf]

nature research

Corresponding author(s): Peng Li

Last updated by author(s): Sep 21, 2022

Reporting Summary

Nature Research wishes to improve the reproducibility of the work that we publish. This form provides structure for consistency and transparency in reporting. For further information on Nature Research policies, see our Editorial Policies and the Editorial Policy Checklist.

Statistics

For all statistical analyses, confirm that the following items are present in the figure legend, table legend, main text, or Methods section.

n/a	Confirmed	
☐	☒	The exact sample size (n) for each experimental group/condition, given as a discrete number and unit of measurement
☐	☒	A statement on whether measurements were taken from distinct samples or whether the same sample was measured repeatedly
☐	☒	The statistical test(s) used AND whether they are one- or two-sided<br>*Only common tests should be described solely by name; describe more complex techniques in the Methods section.*
☒	☐	A description of all covariates tested
☒	☐	A description of any assumptions or corrections, such as tests of normality and adjustment for multiple comparisons
☐	☒	A full description of the statistical parameters including central tendency (e.g. means) or other basic estimates (e.g. regression coefficient) AND variation (e.g. standard deviation) or associated estimates of uncertainty (e.g. confidence intervals)
☐	☒	For null hypothesis testing, the test statistic (e.g. F, t, r) with confidence intervals, effect sizes, degrees of freedom and P value noted<br>*Give P values as exact values whenever suitable.*
☒	☐	For Bayesian analysis, information on the choice of priors and Markov chain Monte Carlo settings
☒	☐	For hierarchical and complex designs, identification of the appropriate level for tests and full reporting of outcomes
☒	☐	Estimates of effect sizes (e.g. Cohen's d, Pearson's r), indicating how they were calculated

Our web collection on statistics for biologists contains articles on many of the points above.

Software and code

Policy information about availability of computer code

Data collection	For flow cytometry, data were collected by BD LSR Fortessa or BD Canto Ⅱ; for bulk RNA-seq, data were collected by BGISEQ-500 (BGI, Wuhan, China); for scRNA-seq, data were collected by a NovaSeq instrument (Illumina, USA).
Data analysis	Graphpad Prism 8 was used for graphing and statistical analysis, FlowJo (V10.4.0) was used for FACS data analysis, R package Seurat (V3.1.5) was used for scRNA-seq data analysis and Cellchat tool (https://github.com/sqjin/CellChat) was used for intercellular comunications analysis base on scRNA-seq data.

For manuscripts utilizing custom algorithms or software that are central to the research but not yet described in published literature, software must be made available to editors and reviewers. We strongly encourage code deposition in a community repository (e.g. GitHub). See the Nature Research guidelines for submitting code & software for further information.

Data

Policy information about availability of data

All manuscripts must include a data availability statement. This statement should provide the following information, where applicable:

- Accession codes, unique identifiers, or web links for publicly available datasets
- A list of figures that have associated raw data
- A description of any restrictions on data availability

All data are included in the Supplementary Information or available from the authors upon reasonable requests, as are unique reagents used in this Article. The Bulk RNA-seq data have been deposited in Sequence Read Archive (SRA) under accession code [SRR18804308-SRR18804322] [https://dataview.ncbi.nlm.nih.gov/object/SRR18804308]. The single cell RNA-seq data have been deposited in SRA under accession code [SRR18750842 and SRR18750843] [https://dataview.ncbi.nlm.nih.gov/object/SRR18750842]. The FACS data have been deposited in in the OMIX, China National Center for Bioinformation under accession code [OMIX001697] [https://ngdc.cncb.ac.cn/omix/release/OMIX001697]. The raw numbers for charts and graphs are available in the Source Data file whenever

possible.

Field-specific reporting

Please select the one below that is the best fit for your research. If you are not sure, read the appropriate sections before making your selection.

☒ Life sciences ☐ Behavioural & social sciences ☐ Ecological, evolutionary & environmental sciences

For a reference copy of the document with all sections, see nature.com/documents/nr-reporting-summary-flat.pdf

Life sciences study design

All studies must disclose on these points even when the disclosure is negative.

Sample size	No sample size calculation was performed. For in vitro studies, a minimum of three biological replicates were assayed. For in vivo experiments, a minimum of 4 animals per treatment group were evaluated to observe statistically significant differences. The magnitude of effect observed was generally large enough to compensate for small sample sizes.
Data exclusions	No data were excluded.
Replication	ALL major experiments (in vitro killing assays, the production of cytokines, in vivo experiments) were repeated more than 3 times and obtain similar results. Bulk RNA-seq have 3 biological replicates, and scRNA-seq was performed once. The numbers of biological replicates for other experiment is indicated in the figure legends, and all obtain similar results.
Randomization	T cells are derived from random donors.<br>Primary NSCLC samples were chosen by surgeons of the hospital, who decided based on treatment guidelines, which patient should undergo surgery and samples were forwarded to the investigators. This sample was taken after formal written consent during an excision surgery from patients with NSCLC, the researchers were blind to any covariate characteristics. This sample was selected because FACS and IHC showed that it expressed both PD-L1 and MSLN at same time after establishing the PDX model.<br>For in vitro studies, identical cell samples were divided into different treatment groups, and no randomization was required. For in vivo studies, allocation of animals was random to each treatment group, and all mice were matched for age and sex within the same experiment. When tumors were established in mice, mice were assigned to treatment groups using randomization base on tumor size, to ensure equal distribution of tumor size between groups.
Blinding	Except for the caliper measurement of the tumor size, all other data collects and analyses were based on objectively measurable data.<br>Caliper measurements were conducted during the whole study by the same investigator who was blinded before assessing tumor size. For other experiments blinding was not strictly required as outcomes were based on objective measures. Experiments were designed to prepare and measure several samples simultaneously in a uniform way. Appropriate controls were always included.

Reporting for specific materials, systems and methods

We require information from authors about some types of materials, experimental systems and methods used in many studies. Here, indicate whether each material, system or method listed is relevant to your study. If you are not sure if a list item applies to your research, read the appropriate section before selecting a response.

Materials & experimental systems

n/a	Involved in the study	
☐	☒	Antibodies
☐	☒	Eukaryotic cell lines
☒	☐	Palaeontology and archaeology
☐	☒	Animals and other organisms
☐	☒	Human research participants
☒	☐	Clinical data
☒	☐	Dual use research of concern

Methods

n/a	Involved in the study	
☒	☐	ChIP-seq
☐	☒	Flow cytometry
☒	☐	MRI-based neuroimaging

Antibodies

Antibodies used	The antibodies used in this research included<br>anti-human CD3-PE/cyanine 7 (clone: UCHT1, 300420, Biolegend),<br>anti-human CD4-APC (clone: OKT4, 317416, Biolegend),<br>anti-human CD4-APC/cyanine 7 (clone: OKT4, 317418, Biolegend),<br>anti-human CD8a-PE (clone: HIT8a, 344706, Biolegend),<br>anti-human CD8a-PerCP/Cyanine 5.5 (clone: HIT8a, 300924, Biolegend),<br>anti-human CD28-APC (clone: CD28.2, 17-0289-42, Invitrogen),<br>anti-human CD28-APC/cyanine7 (clone: CD28.2, 302966, Biolegend),

anti-human 4-1BB-PE (clone: 4B4-1, 309804, Biolegend),
anti-human CD19-APC (clone: HIB19, 302212, Biolegend),
anti-human CD25-PE (clone: BC69, 302606, Biolegend),
anti-human CD69-APC/cyanine 7 (FN50, 310910, Biolegend),
anti-human PD-L1-APC (clone: M1H2, 393610, Biolegend),
anti-human MSLN-APC (clone: 420411, FAB32652A, R&D),
and anti-human IL13 (clone: 85BRD, 12-7136-42, Invitrogen).
anti-human CD70-PE/cyanine7 (clone: 113-16, 355112, Biolegend),
anti-human CD27-PE (clone: M-T271, 356406, Biolegend),
anti-human PD-L1 (MK-3475, A2004, Selleck),
anti-human PD-1 (MPDL3208A, A2005, Selleck),
anti-human IL2 (MQ1-17H12, 500301, Biolegend),
anti-human IFN-γ (NIB42, 16-7318-81, ThermoFisher)
anti-human CD70 (BU69, ab213102, Abcam)

Validation

All antibodies were commercially available, and used only on species for which they have been validated by the manufacturers or published research.
anti-human CD3-PE/cyanine 7 (mouse, FACS), https://www.biolegend.com/en-us/products/pe-cyanine7-anti-human-cd3-antibody-3070;
anti-human CD4-APC (mouse, FACS), https://www.biolegend.com/en-us/products/apc-anti-human-cd4-antibody-3657;
anti-human CD4-APC/cyanine 7 (mouse, FACS), https://www.biolegend.com/en-us/products/apc-cyanine7-anti-human-cd4-antibody-3658;
anti-human CD8a-PE (mouse, FACS), https://www.biolegend.com/en-us/products/pe-anti-human-cd8-antibody-6247;
anti-human CD8a-PerCP/Cyanine 5.5 (mouse, FACS), https://www.biolegend.com/en-us/products/percp-cyanine5-5-anti-human-cd8a-antibody-5615;
anti-human CD28-APC (mouse, FACS), https://www.thermofisher.cn/cn/zh/antibody/product/CD28-Antibody-clone-CD28-2-Monoclonal/17-0289-42;
anti-human CD28-APC/cyanine7 (mouse, FACS), https://www.biolegend.com/en-us/products/apc-cyanine7-anti-human-cd28-antibody-17216;
anti-human 4-1BB-PE (mouse, FACS), https://www.biolegend.com/en-us/products/pe-anti-human-cd137-4-1bb-antibody-1510;
anti-human CD19-APC (mouse, FACS), https://www.biolegend.com/en-us/products/apc-anti-human-cd19-antibody-715;
anti-human CD25-PE (mouse, FACS), https://www.biolegend.com/en-us/products/pe-anti-human-cd25-antibody-616;
anti-human CD69-APC/cyanine 7 (mouse, FACS), https://www.biolegend.com/en-us/products/apc-anti-human-cd69-antibody-1674;
anti-human PD-L1-APC (mouse, FACS), https://www.biolegend.com/en-us/products/apc-anti-human-cd274-b7-h1-pd-l1-antibody-16138;
anti-human MSLN-APC (Rat, FACS), https://www.rndsystems.com/cn/products/human-mesothelin-apc-conjugated-antibody-420411_fab32652a;
and anti-human IL13 (Rat, FACS), https://www.thermofisher.cn/cn/zh/antibody/product/IL-13-Antibody-clone-85BRD-Monoclonal/12-7136-42;
anti-human CD70-PE/cyanine7 (mouse, FACS), https://www.biolegend.com/en-us/products/pe-cyanine7-anti-human-cd70-antibody-14533;
anti-human CD27-PE (mouse, FACS), https://www.biolegend.com/en-us/products/pe-anti-human-cd27-antibody-8371;
anti-human PD-L1 (CHO cells, Cell and animal experiments), https://www.selleck.cn/products/atezolizumab.html;
anti-human PD-1 (CHO cells, Cell and animal experiments), https://www.selleck.cn/products/pembrolizumab.html;
anti-human IL2 (Rat, ELISA, CyTOF, IHC, IP, IC, Neu), https://www.biolegend.com/en-us/products/purified-anti-human-il-2-antibody-1352;
anti-human IFN-γ (mouse, WB, ELISA, Neu, FN, IA, ICC/IF), https://www.thermofisher.cn/cn/zh/antibody/product/IFN-gamma-Antibody-clone-NIB42-Monoclonal/16-7318-81;
anti-human CD70 (mouse, ICC/IF, Flow Cyt, IHC-Fr, IP, Functional Studies); https://www.abcam.cn/cd70-antibody-bu69-bsa-and-azide-free-ab213102.html.

Eukaryotic cell lines

Policy information about cell lines

Cell line source(s)

HEK-293T, H460, HeLa, NALM6 and K562 cell lines were purchased from ATCC and stocked in our lab. BGC823 cell line was purchased from Shangcheng Beina Chuanglian Biological Technology Co., LTD (BNCC352070) and stocked in our lab.

Authentication

We confirmed these cell lines through Short Tandem Repeat (STR) test.

Mycoplasma contamination

All cell lines were tested negative for mycoplasma contamination.

Commonly misidentified lines
(See ICLAC register)

BGC823 cell line was used as the target cells of CARPAz T cells in this research, as it highly expresses PSCA (prostate stem cell antigen). This cell line is on the list of "Known misidentified cell lines". We have authenticated this cell line by STR (short tandem repeat) method. The result showed that BGC823 cell line was an uncontaminated cell line, but the STR typing of this cell line was 100% matches with HeLa cells. Therefore, we have replaced BGC823-GL cells in the Fig. 7a-e and supplemental Fig. 8e with HeLa-GL cells, respectively.

Animals and other organisms

Policy information about studies involving animals; ARRIVE guidelines recommended for reporting animal research

Laboratory animals	All mice used in these studies were aged 6-8 weeks, equivalent numbers of male and female NOD-SCID-IL2Rg-/- (NSI) mice. All experimental mice were co-housed within specific pathogen-free (SPF)-grade cages and provided autoclaved food and water, with a 12 h light/dark cycle and a temperature range of 21-27℃ with 40-60% humidity.
Wild animals	This study does not involve wild animals.
Field-collected samples	This study does not involve field-collected samples
Ethics oversight	Animal experiments were performed in the Laboratory Animal Center. All animal experiments were performed based on an animal protocol approved by the relevant institutional animal care and use committee (IACUC) of Guangzhou Institutes of Biomedicine and Health.

Note that full information on the approval of the study protocol must also be provided in the manuscript.

Human research participants

Policy information about studies involving human research participants

Population characteristics	Human PBMCs from healthy donors were obtained from cooperative organization, the researchers were blind to any covariate characteristics.<br>The primary NSCLC sample was from a 59 years old male low differentiated squamous cell carcinoma, Ⅲa phase, without EGFR mutation. This sample was taken after formal written consent during an excision surgery from patients with NSCLC, the researchers were blind to any covariate characteristics.
Recruitment	T cells are derived from random donors.<br>Patient derived tumor samples were chosen by surgeons of the hospital, who decided based on treatment guidelines, which patient should undergo surgery and samples were forwarded to the investigators.
Ethics oversight	The primary NSCLC sample was from a 59 years old male low differentiated squamous cell carcinoma, Ⅲa phase, without EGFR mutation. This volunteer provided written informed consent, and uses of human material have been approved by the Research Ethics Board of Guangzhou Institutes of Biomedicine and Health.

Note that full information on the approval of the study protocol must also be provided in the manuscript.

Flow Cytometry

Plots

Confirm that:

- [x] The axis labels state the marker and fluorochrome used (e.g. CD4-FITC).
- [x] The axis scales are clearly visible. Include numbers along axes only for bottom left plot of group (a 'group' is an analysis of identical markers).
- [x] All plots are contour plots with outliers or pseudocolor plots.
- [x] A numerical value for number of cells or percentage (with statistics) is provided.

Methodology

Sample preparation	Cell-surface staining was performed by pelleting cells and resuspending them in 50 µl of FACS buffer (2% FBS in PBS) with antibodies for 30 min on ice in the dark. For intracellular staining, cells were fixed with Phosflow™ Fix Buffer Ⅰ (BD, 557870) and permeabilized with Phosflow™ Perm Buffer Ⅲ (BD, 558050). Peripheral blood, spleen and tumor samples from xenograft mice were treated with red blood cell lysis buffer (BioLegend, 420301) before staining. Cells were washed with FACS buffer before analysis.
Instrument	Flow cytometry was performed on a BD LSR Fortessa and BD Canto Ⅱ.
Software	The data were analyzed using FlowJo software (V10.4.0).
Cell population abundance	Cell populations of cells used for Bulk RNA-seq or scRNA-seq were validated for purity by a post-sort analysis by FACS, and cell frequencies more than 90%.
Gating strategy	Every flow cytometry analysis was initiated as follows: FSC-A/SSC-A to gate the lymphocyte population; FSC-A/FSC-H to select single cells; followed by the gating as described in the figures.

- [x] Tick this box to confirm that a figure exemplifying the gating strategy is provided in the Supplementary Information.
